# Influence of Physical Exercise on the Rehabilitation of Volumetric Muscle Loss Injury Reconstructed with Autologous Adipose Tissue

**DOI:** 10.3390/jfmk9040188

**Published:** 2024-10-08

**Authors:** Maria E. Lopez-Espejo, Ignacio Jimena, Maria-Jesus Gil-Belmonte, Jose-Luis L. Rivero, Jose Peña-Amaro

**Affiliations:** 1 Department of Morphological Sciences, Section of Histology, Faculty of Medicine and Nursing, Maimonides Institute for Biomedical Research IMIBIC, Reina Sofía University Hospital, University of Cordoba, 14004 Cordoba, Spain; lopezespejo.me@gmail.com (M.E.L.-E.); cm1jimei@uco.es (I.J.); mjgilbelmonte@gmail.com (M.-J.G.-B.); 2 Department of Pathology, Torrecardenas University Hospital, 04009 Almeria, Spain; 3 Muscular Biopathology Laboratory, Department of Comparative Anatomy and Pathological Anatomy and Toxicology, Faculty of Veterinary Medicine, University of Cordoba, 14014 Cordoba, Spain; an1lorij@uco.es

**Keywords:** skeletal muscle, muscle regeneration, exercise, rehabilitation, volumetric muscle loss, adipose tissue

## Abstract

Background: In volumetric muscle loss (VML) injuries, spontaneous muscle regeneration capacity is limited. The implantation of autologous adipose tissue in the affected area is an option to treat these lesions; however, the effectiveness of this therapy alone is insufficient for a complete recovery of the damaged muscle. This study examined the influence of treadmill exercise on the rehabilitation of VML injuries reconstructed with autologous adipose tissue, as a strategy to counteract the limitations of spontaneous regeneration observed in these injuries. Methods: Forty adult male Wistar rats were divided into eight groups of five individuals each: normal control (NC), regenerative control (RC), VML control (VML), VML injury reconstructed with fresh autologous adipose tissue (FAT), exercise-rehabilitated control (RNC), exercise-rehabilitated regenerative control (RRC), exercise-rehabilitated VML injury (RVML), and exercise-rehabilitated VML injury reconstructed with fresh autologous adipose tissue (RFAT). Histological and histochemical staining techniques were used for the analysis of structural features and histomorphometric parameters of the tibialis anterior muscle. Grip strength tests were conducted to assess muscle force. Results: Exercise rehabilitation decreased the proportion of disoriented fibers in RFAT vs. FAT group. The percentage of fibrosis was significantly higher in FAT and RFAT groups versus NC and RNC groups but did not vary significantly between FAT and RFAT groups. Overall, muscle grip strength and fiber size increased significantly in the exercise-rehabilitated groups compared to control groups. Conclusions: To conclude, rehabilitation with physical exercise tended to normalize the process of muscle repair in a model of VML injury reconstructed with fresh autologous adipose tissue, but it did not reduce the intense fibrosis associated with these injuries.

## 1. Introduction

Skeletal muscle is one of the tissues with the greatest regenerative capacity after injury [1,2]. Nevertheless, in the context of muscle injuries with volumetric loss (“volume muscle loss”—VML), where there is a substantial reduction in muscle mass (such as in traffic accidents, in major catastrophes, or after tumor removal surgeries), the physiological capacity of muscle repair is overwhelmed [3,4]. The percentage of muscle mass from which the injury is irreparable has been estimated at ~15% [5]. In these circumstances, a greater deposition of collagen and chronic inflammatory infiltrate are observed, with the consequent structural and functional muscle deterioration. Moreover, this is favored by the insufficient number of myogenic progenitors needed to fill the area (practically null), as well as by the almost total destruction of the extracellular matrix (ECM), local nerves, and blood vessels [3].

Currently, the treatment of choice to repair these injuries is the transplantation of autologous muscle grafts [6]. But the high rate of graft failure together with the remaining morbidity in the donor muscle area [7] make it an incomplete measure to achieve the desired functional results. To date, several treatment options have been studied and tested to restore functionality through tissue engineering techniques, combining the use of stem cells, growth factors, and biomaterials [3], as well as the use of physical therapies, including post-injury physical exercise regimens [8]. Altogether, these strategies have shown an improvement in VML lesions compared to those that have not received any therapeutic intervention. However, there is still controversy as to whether these options substantially improve muscle function [9].

Recently, the study of the usefulness of mesenchymal stem cells from adipose tissue as precursors of different tissues, including skeletal muscle, has been intensified, reaching great prominence in the field of translational medicine [10,11]. For these cells to act as myogenic progenitors, an adequate environment is needed to promote their differentiation towards the myogenic phenotype [4]. Thus, one of the experimental approaches recently tested to treat VML lesions has been the implantation of autologous adipose tissue directly in the affected area [12]. In that study, a group of rats with an induced VML defect in the muscle tibialis anterior in which autologous adipose tissue had been implanted was compared with another group of sham-operated control rats with the same VML defect in the m. tibialis anterior in which frozen autologous adipose tissue was implanted (three repetitive cycles of direct immersion in liquid nitrogen performed in a total period of three minutes) in order to achieve total destruction of the cells and was used as an appropriate comparison control group. At 60 days post-intervention, and compared to the sham-operated group, a greater fraction of the implanted autologous adipose tissue was replaced by muscle tissue in the adipose graft group. This intervention showed the neoformation of muscle fibers; nonetheless, the newly formed muscle carried important structural abnormalities such as the presence of disoriented muscle fibers or a high percentage of fibrosis. Therefore, it is necessary to design new therapeutic strategies for the efficient management of VML lesions reconstructed with autologous adipose tissue to obtain a reconstructed muscle as similar as possible to normal muscle. Previously, different studies had shown the effect of a physical exercise rehabilitation plan on VML injuries without [8] and with [13] subsequent reconstruction using tissue engineering techniques, demonstrating benefits in muscle remodeling and functionality. Exercise participates in the process known as “mechanotransduction”, acting as a mechanical stimulus on the muscle that causes variations in both cellular and extracellular expression of muscle fibers, and, therefore, muscular changes at the biochemical and structural level [14].

Upon this background, this study tested the hypothesis that the application of a re-habilitation plan through physical exercise benefits the histoarchitecture of newly formed skeletal muscle in VML lesions reconstructed with autologous adipose tissue, counteracting the structural alterations associated with these lesions. The main objective was to study the impact of physical exercise on skeletal muscle histoarchitecture in VML lesions reconstructed with autologous adipose tissue. For this goal, the (i) cellular (cell size, fibers with internal nuclei, and spatial orientation of myofibers), (ii) extracellular (endo and perimisial fibrosis), and (iii) functional (muscular grip strength) characteristics of the newly formed muscle were specifically analyzed.

## 2. Materials and Methods

### 2.1. Animals and Study Design

A cohort of 40 adult male Wistar rats (3 months old), weighing 437 ± 40 g (mean ± SD, range, 365–550 g), were used in this study. All animals were kept under controlled temperature and lighting conditions, and with ad libitum access to food and water. All procedures were carried out in accordance with Directive 2010/63/EU of the Council and European Parliament that governs the protection, welfare, and use of animals for scientific purposes. This study was approved by the General Directorate of Agricultural and Live-stock Production of the Junta de Andalucia (Ref. 07/04/2021/043), the Ethics Committee for Animal Experimentation of the University of Cordoba, and the Research Ethics Committee of the Province of Cordoba.

By using a basic computer application, the animals were randomly allotted into 8 groups of 5 rats each (Figure S1): (i) normal control (NC), formed by baseline rats without any intervention; (ii) regenerative control (RC), composed of rats that had a myotoxin injected into their tibialis anterior (TA) muscle and were used as a control of the normal regenerative process; (iii) a control group with VML injury (VML); and (iv) a group with VML injury reconstructed with fresh autologous adipose tissue (FAT). These same four groups were duplicated with rats subjected to a 5-week period of exercise rehabilitation on a treadmill, completing the following groups: (v) exercise-rehabilitated normal control (RNC); (vi) exercise-rehabilitated regenerative control (RRC); (vii) exercise-rehabilitated VML injury (RVML); and (viii) exercise-rehabilitated VML injury reconstructed with fresh autologous adipose tissue (RFAT). Although toxin-induced muscle injuries are regenerative, the tissue response to injury is very different to that of VML, particularly in very extensive VML injuries constituting >15% of muscle weight [5]. Therefore, the use of a toxin-induced muscle injury group in the present study was included to explore the effect of exercise on muscle regenerative capacity and not as an appropriate control for VML injuries.

All experimental interventions were conducted in a blinded manner, ensuring that each operator did not have access to each individual’s identification control. For this purpose, each animal or test sample was identified with a control code unrelated to the experimental group to which it belonged.

### 2.2. Surgical and Experimental Procedures

**Figure 1:**
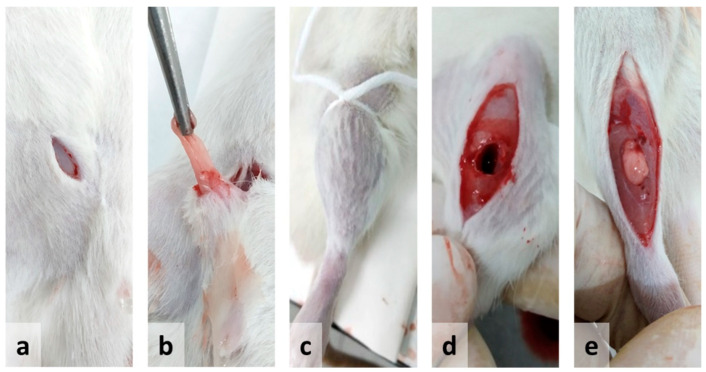
Illustration of the experimental procedure for the implantation of autologous adipose tissue in muscle injury due to volumetric loss (volumetric muscle loss—VML) used in the present study: (**a**) incision in the inguinal area; (**b**) extraction of adipose tissue; (**c**) tourniquet; (**d**) volumetric defect in the tibialis anterior muscle; (**e**) adipose tissue inserted in the area with the volumetric defect.

All surgical procedures were performed under general anesthesia and aseptic conditions. To induce muscle degeneration–regeneration in the RC and RRC groups, 100 µL of a solution of 2% mepivacaine hydrochloride 2% (Scandinibsa; Inibsa, Barcelona, Spain) was injected through the skin into the belly of the TA muscle. To induce volumetric loss injury in the VML, RVML, FAT, and RFAT groups, after exposing the TA muscles, a fragment of the muscle belly, weighing between 200 and 300 mg (38% of muscle weight), was removed bilaterally by using a sterile 6 mm in diameter and 5 mm in length punch. The muscle excision site was standardized at the geometric center of the muscle belly. Afterwards, subcutaneous adipose tissue, obtained from the inguinal region of the same animals, was implanted in the VML defect area of both muscles (Figure 1). During the procedure, a tourniquet was applied for 3–4 min proximal to the excision site only in the VML groups, to reduce bleeding at the time of removal and subsequent transplantation of adipose tissue. A hemostatic sponge (standard Gellita-Spon ^®^, Gellita Medical GmbH, Eberbach, Germany) was also used to prevent bleeding. The adipose tissue fragments, after extraction from the inguinal region of the same animal, were kept in saline until implantation. The amount of transplanted tissue was like that of the previously extracted muscle. A fraction of adipose tissue was used for in vitro determinations (e.g., adiponectin) not included in the present study. After cleaning, disinfecting with 10% povidone iodine, and suturing with prolene 6-0 the intervened areas (fascia and skin), ceftriaxone diluted in the drinking water was administered preventively to each rat (20 mg/kg BW) only in the VML, RVML, FAT, and RFAT groups.

**Figure 2:**
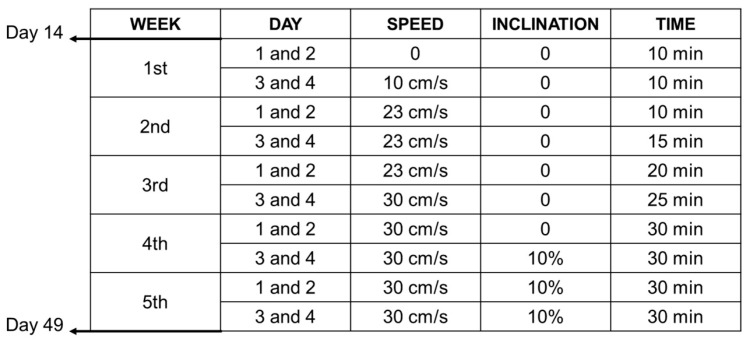
Experimental design. Synopsis of the exercise rehabilitation protocol tested in the present study.

Starting on day 14, the animals from the RNC, RRC, and RFAT groups performed aerobic physical exercise sessions for 5 weeks, 4 days a week, following a prescribed protocol (Figure 2). All these exercises were performed on a specific treadmill for experimental animals (Treadmill LE8710RTS, Panlab, Barcelona, Spain). This rehabilitation intervention ended on day 49, with the animals remaining without exercise for up to 60 days. To ensure that all rats completed this exercise program, the treadmill has a shock grid to force the animals to complete the exercise, but the use of this system was occasional.

Muscle grip strength was determined before (day 14) and after (day 60) completion of the rehabilitation exercise program for the bilateral hind limbs using a “grip strength meter” (Model GS3, Bioseb’s Grip Strength Test, Vitrolles, France). The grip strength test was performed by grasping the rat by the tail and having it climb an inclined rack equipped with a thin bar attached to a dynamometer to record force [15,16]. The force measurements were averaged from three trials. The test was performed in a blinded manner by an investigator (I.J.) who was unaware of which experimental group each rat belonged to. After 60 days, all rats were sacrificed under deep general anesthesia via intraperitoneal injection of 50 mg/kg bw sodium thiopental. Immediately afterwards, bilateral TA muscles were extracted and processed for microscopic analysis.

### 2.3. Sample Processing

**Figure 3:**
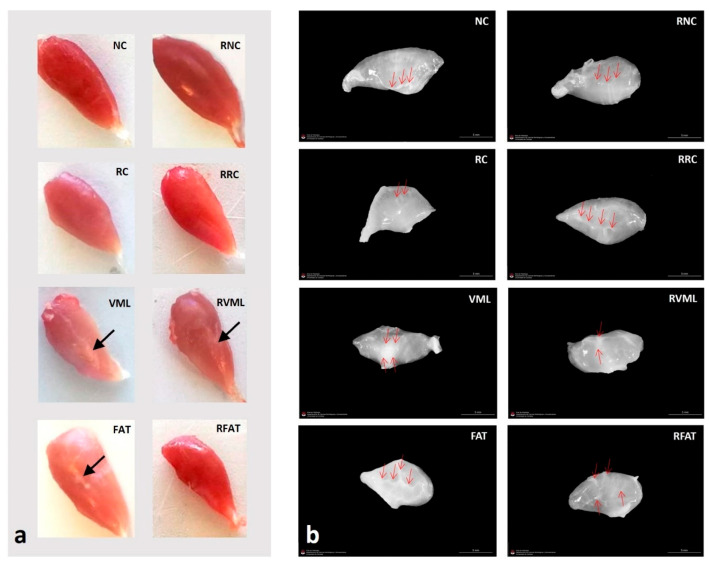
(**a**) Representative gross appearances of tibialis anterior muscles in the different experimental groups. Note a thin scar (arrow) in the central area of the VML, RVML, and FAT muscles, and how the RFAT muscle is thinner and smaller. (**b**) Representative gross appearances of decellularized matrices of tibialis anterior muscles in the different experimental groups. Red arrows show areas with a higher content of connective tissue that may correspond to either intramuscular tendons or fibrotic areas. Note how the matrices of the NC, RC, VML, and FAT groups are denser compared to those of the equivalent rehabilitated groups. NC: normal control; RC: regenerative control; VML: volumetric muscle loss; FAT: VML with fresh autologous adipose tissue implantation; RNC: exercise-rehabilitated normal control; RRC: exercise-rehabilitated regenerative control; RVML: exercise-rehabilitated VML lesion RFAT: exercise-rehabilitated VML lesion with fresh autologous adipose tissue implant.

Muscle samples were frozen in isopentane pre-cooled in liquid nitrogen, obtaining cross-sections of 8 µm thickness in a cryostat (Leica CM1850 UV, Leica Microsystems, Nussloch, Germany) at −20 °C. The muscle samples used for microscopic analyzes in the animals with VML injury (VML, RVML, FAT, and RFAT groups) were obtained from the same central area of the muscle in which the initial injury scar was observed (see Figure 3a). Thus, the site where the adipose tissue was autografted was evaluated.

To analyze the ECM, other sample fragments were decellularized following the protocol described in Sleboda and coworkers [17].

### 2.4. Histological and Histochemical Techniques

For the microscopic study, the classic protocols described by Dubowitz and Sewry were followed [18]. For the general morphological study, the samples were stained with hematoxylin-eosin (H-E) and Van Gieson’s trichrome (VGT). As a histochemical technique, the nicotinamide adenine dinucleotide tetrazolium reductase reaction (NADH-tr) was included to visualize myofibers’ mitochondrial content.

### 2.5. Histomorphometry

Photomicrographs of the different stains on serial cross-sections of the samples were obtained with a Nikon Eclipse E1000 microscope (Nikon, Tokyo, Japan) equipped with a Sony DXC-990P color video camera (Sony, Tokyo, Japan).

The images for morphometric analysis were examined with the image analysis soft-ware ImageJ^®^ v.2.3.0/1.53q (Rasband, W.S, National Institutes of Health, Bethesda, MD, USA). Five fields of each H-E-stained sample were photographed at 40× magnification. A total of 203 ± 52 fibers (mean ± SD; range, 87–319 fibers) were analyzed for each of the 5 fields. The following parameters were calculated in each field: number of fibers, mean fiber cross-sectional area (CSA), lesser fiber diameter (LFD), number of fibers with internal nuclei, number of disoriented fibers, form factor, and percentage of the muscle area occupied by connective tissue (fibrosis). The lesser (or minimal) fiber diameter is defined as the maximum diameter across the lesser aspect of the fiber and is designed to overcome the distortion that occurs either when the fiber is cut obliquely or when there is kinking of the muscle fiber, both common artifacts in muscle samples that alter considerably other morphometric measurements of fiber size such as the CSA [18]. Disoriented fibers are those that, in cross section, do not show a normal spatial distribution, i.e., they do not show a polygonal outline but irregular edges (concave or convex) and an oblique cross section. In addition to the CSA and LFD of each muscle fiber, information was also obtained on the maximum fiber diameter (MFD). The relationship between the maximal and minimal diameters of a fiber provides information on the obliquity of the cut, assuming that the computer-simulated elliptical structure of the polygonal myofibers has maximal and minimal diameters that are identical (i.e., a circle) when the fiber is cut perpendicular to its longitudinal axis. On this basis, a form factor (FF) to identify regular or irregular fibers was calculated according to the formula of Larsson and Skogsberg [19]:

(1) $$FF=\frac{CSA}{(\frac{\pi }{4})\times LFD\times MFD}$$

The FF for a circle or an ellipse is equal to one and for irregular structures < 1.

### 2.6. Statistics

The sample size and power of the hypothesis test were estimated via power analysis and interval estimation. Overall, accepting a α-risk of 0.05 and a β-risk of 0.2 in a two-tailed test, 8 rats/group were necessary to recognize as statistically significant a difference greater than 1 SD unit between groups, assuming a common deviation of 20% of the mean values and predicting a dropout rate of 5%. Due to reasons of animal welfare, this sample size estimation was not applied in the present study, being reduced to 5 animals/group. In all the analyses carried out, 5 animals per group were included, with no exclusions.

Data were analyzed using Statistics 9.0 for Windows (StatFort I, Statistica, Data Software System: www.statsoft.com accessed on 5 October 2024). Muscle sample was the unit of analysis for the present data set. The average of 5 fields was obtained from each sample, expressing the results of each group as mean (SD). For each variable, a normality test (Shapiro–Wilk test) was performed to select the indicated statistical test (parametric or non-parametric). Quantitative variables with normal distribution (body weight and muscle weight) were analyzed using one-way analysis of variance (one-way ANOVA); and comparisons between each pair of 2 × 2 groups were made using Tukey’s test. Quantitative variables without normal distribution (grip muscle strength at 60 days, percentage of fibers with internal nuclei, CSA, LFD, and area of fibrosis) were compared between groups using the Kruskal–Wallis h test; and comparisons between groups 2 to 2 were made with Mann–Whitney U tests. To assess possible variations in grip muscle strength within the groups undergoing rehabilitation, before (day 14) and after (day 60) the intervention, the Student’s t-test for paired data was used. In addition, to compare the percentages of disoriented muscle fibers between the FAT and RFAT groups, a Student’s t-test for independent variables was performed. All tests were conducted at a significant level of 5% (*p* < 0.05).

## 3. Results

Macroscopically, muscles from all groups showed normal appearances. With respect to decellularized matrices, rehabilitated muscles showed lower density, and central scars were frequently observed around the old VML lesion areas in VML, FAT, RVML, and RFAT groups (Figure 3).

**Figure 4:**
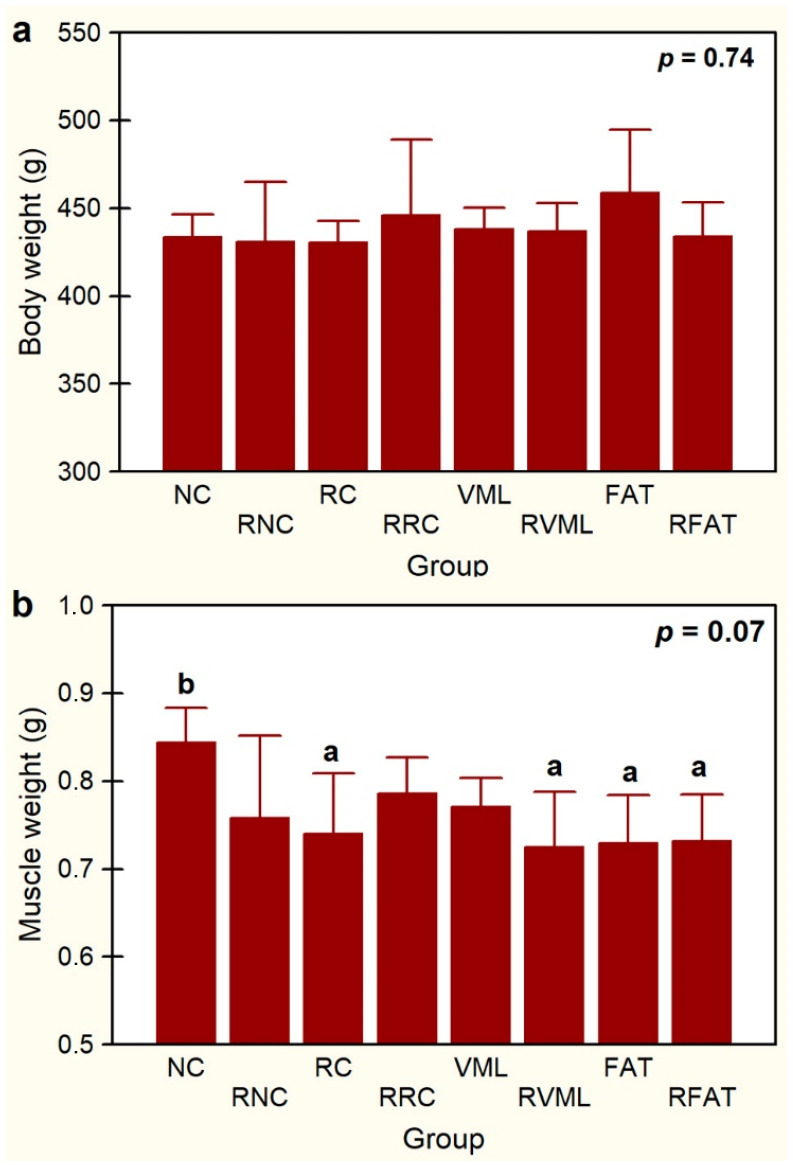
Body weight (**a**) and muscle weight (**b**) in experimental groups. Data expressed as mean ± SD of 5 rats per group. *p* value of a one-way ANOVA between groups is showed. Means with different lowercase letters are statistically significant (*p* < 0.05, at least). See Figure 3’s legend for keys.

No statistically significant differences in body weight were observed among groups (Figure 4a). A non-significant trend (*p* < 0.1) for differences in muscle weight was observed (Figure 4b), being significantly higher in NC rats than in RC, RVML, FAT, and RFAT rats (12%, *p* < 0.05).

**Figure 5:**
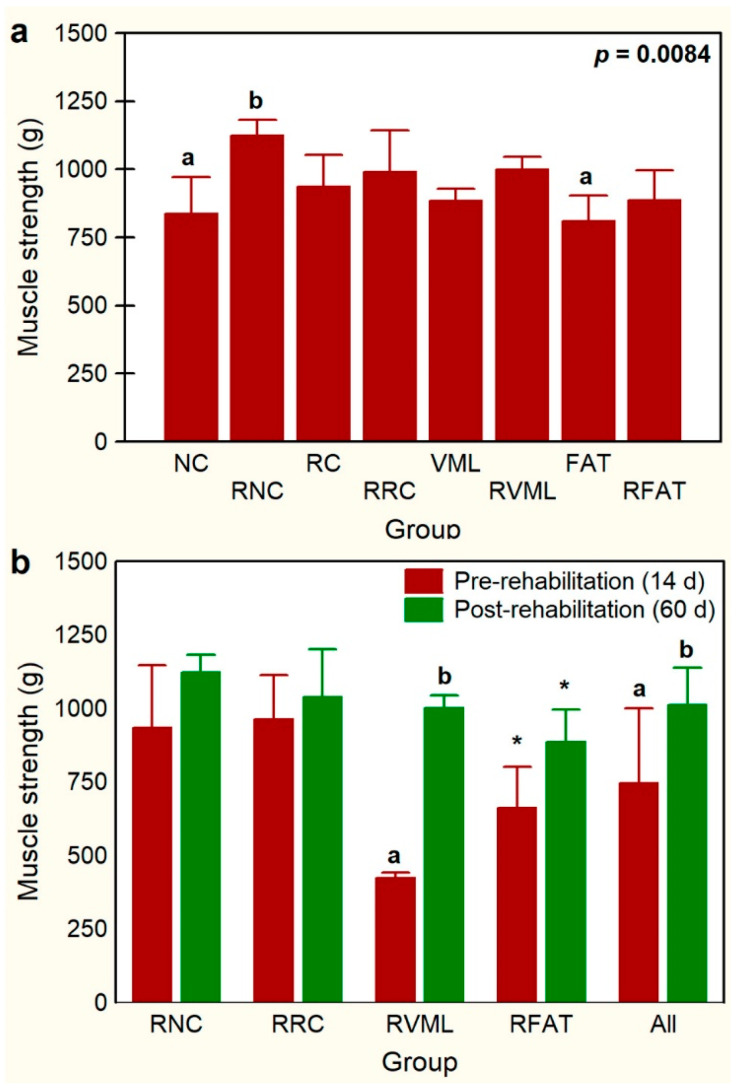
Comparisons of muscle grip strength of pelvic limbs among experimental groups (**a**) and before (day 14) and after (day 60) a program of exercise-based rehabilitation (**b**). Data are expressed as mean ± SD of 5 rats per group. *p* value of a nonparametric Kruskal–Wallis h test followed by a Mann–Whitney U test for 2 × 2 comparisons between groups is shown in (**a**). Means with different lowercase letter are statistically significant (*p* < 0.05 at least). In panel (**b**), for each experimental group, means with different lowercase letter are statistically significant (*p* < 0.05 at least) according to a paired Student’s t test between pre- and post-rehabilitation; * denotes a not significant trend (*p* < 0.1) between pre- and post-rehabilitation groups. See Figure 3’s legend for keys.

Animals’ functional evaluation, carried out 14 days post-injury, was normal, presenting good mobility without apparent weakness, paresis, or abnormalities in the limbs’ positioning. An improvement in muscle grip strength observed after 5 weeks of rehabilitation was significantly greater in the RNC group rats than in both the NC (25%, *p* = 0.03) and FAT (28%, *p* = 0.015) groups (Figure 5a). Similarly, in all the rats of the four rehabilitated groups (n = 20 rats), the muscle grip strength of pelvic limbs increased significantly (*p* = 0.00) by 36.1% at day 60 (1014 ± 132 g) compared to day 14 (745 ± 267 g). This increase in pelvic limb strength was mostly associated with the RVML group of rats, where it increased by 136% after the rehabilitation period (*p* = 0.000) (Figure 5b). Exercise rehabilitation also tended to increase pelvic limb strength in the RFAT group rats, which increased by 34% (*p* = 0.085) (Figure 5b). However, when comparing each pair of the other two groups individually (RNC and RRC), before and after rehabilitation, no statistically significant differences were observed (Figure 5b).

**Figure 6:**
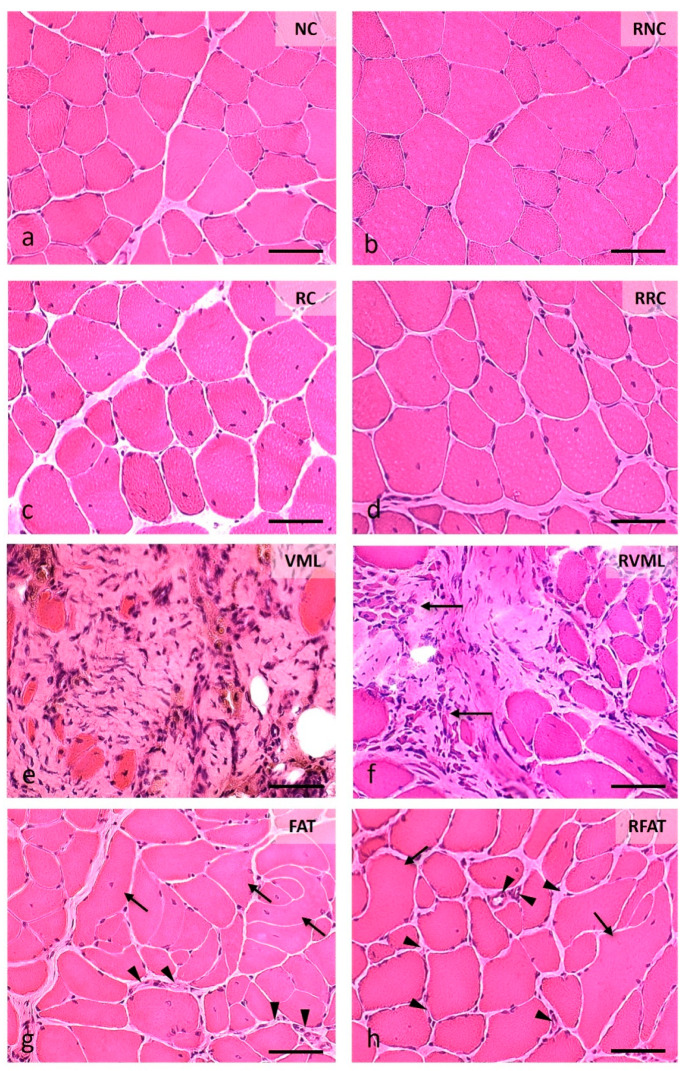
Muscle fiber morphology at 60 days in cross-sections stained with Hematoxylin-Eosin (H-E). (**a**) Normal morphology of muscle fibers with nuclei in a peripheral position and very small perimysial and endomysial spaces. (**b**) Image of normal-looking muscle fibers, although larger than those in the previous image, and a slight presence of the endomysium and perimysium. (**c**) Area showing muscle fibers with slightly rounded contours, normal color, and abundant internal nuclei, as well as increased endomysial and perimysial spaces. (**d**) Image like the previous one, although the size of the muscle fibers is slightly larger. (**e**) Marked fibrosis and occasional small muscle fibers. (**f**) Central fibrotic area surrounded by muscle fibers of variable size, many with internal nuclei; on the left, extremely small groups of muscle fibers stand out (arrows). (**g**) Image with excessive variability in the size of muscle fibers, many of them with internal nuclei, and a few fibers clearly disoriented and cut obliquely or longitudinally (arrows); the perimysium and endomysium are increased, and the vascular network is clearly perceived (arrowheads). (**h**) Histological pattern like the previous one, although with a lower number of disoriented muscle fibers (arrows) and myofibers with internal nuclei; the endomysium is also enlarged and with an apparent higher number of blood vessels (arrowheads). Size scale: 50 µm. See Figure 3’s legend for keys.

Variations in muscle histoarchitecture among groups were evident at 60 days (Figure 6, Figures S2 and S3). A lower number of both disoriented muscle fibers (defined in “Methods”) and cells with internal nuclei, as well as a higher number of vascular lumens, were observed in the RFAT group compared to non-rehabilitated groups.

**Figure 7:**
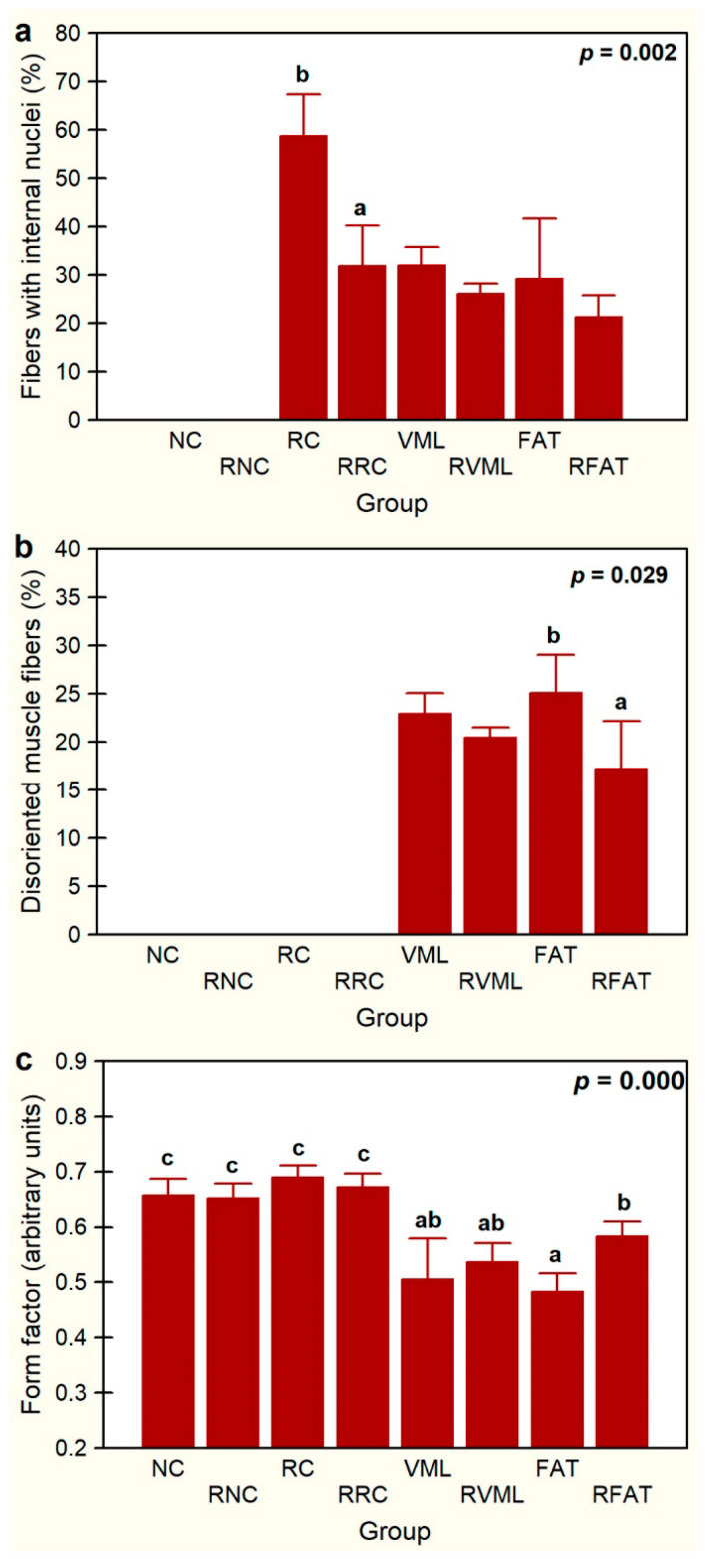
Percentages of fibers with internal nuclei (**a**), percentages of disoriented muscle fibers (**b**), and average form factor (**c**) in the experimental groups. Data are expressed as mean ± SD of 5 rats per group. *p* values of a nonparametric Kruskal–Wallis h test followed by a Mann–Whitney U test for 2 × 2 comparisons between groups (percentage of myofibers with internal nuclei and form factor) and Student’s t test (percentage of disoriented). Means with different lowercase letters are statistically significant (*p* < 0.05, at least). See Figure 3’s legend for keys.

The percentage of fibers with internal nuclei varied significantly among experimental groups (*p* = 0.002, Figure 7a). These fibers were absent in both the NC and RNC groups. The percentage of these fibers decreased by 46% in the RRC group compared to the RC group (*p* = 0.001). The percentage of fibers with internal nuclei was also 27% lower in the RFAT group than in the FAT control group, but this difference only showed a marginal trend that did not reach the level of statistical significance (*p* = 0.059). A similar non-significant trend for the reduction in this fiber percentage was observed between the VML and RVML groups.

The presence of disoriented muscle fibers was only recorded in the groups of rats that suffered volume loss injury (VML, RVML, FAT, and RFAT), as these fibers were not observed in the remaining control or rehabilitated groups (Figure 7b). Importantly, the percentage of muscle fibers with spatial disorientation was significantly lower (31%) in the RFAT group than in the FAT group (*p* = 0.029). This percentage was also 10% lower in the RVML group than in the VML group, but this difference did not reach the level of statistical significance pre-established in this study (*p* = 0.057). Myofiber form factor, a measure of the circularity of the muscle cell cross section, was 28% lower in rats transplanted with autologous adipose tissue than in control rats (*p* = 0.0006), but this factor was significantly higher (21%) in the RFAT group than in the FAT group (*p* = 0.001) (Figure 7c). It was also significantly lower in the volumetric muscle loss groups compared to normal and regenerated controls (21%, *p* = 0.007), but this factor did not vary significantly between VML and RVML rats.

Overall, these data indicate that the exercise-rehabilitation favored the migration of the internal nuclei towards the periphery of the cell and recovery of the spatial distribution pattern and cross-sectional morphology of muscle fibers.

**Figure 8:**
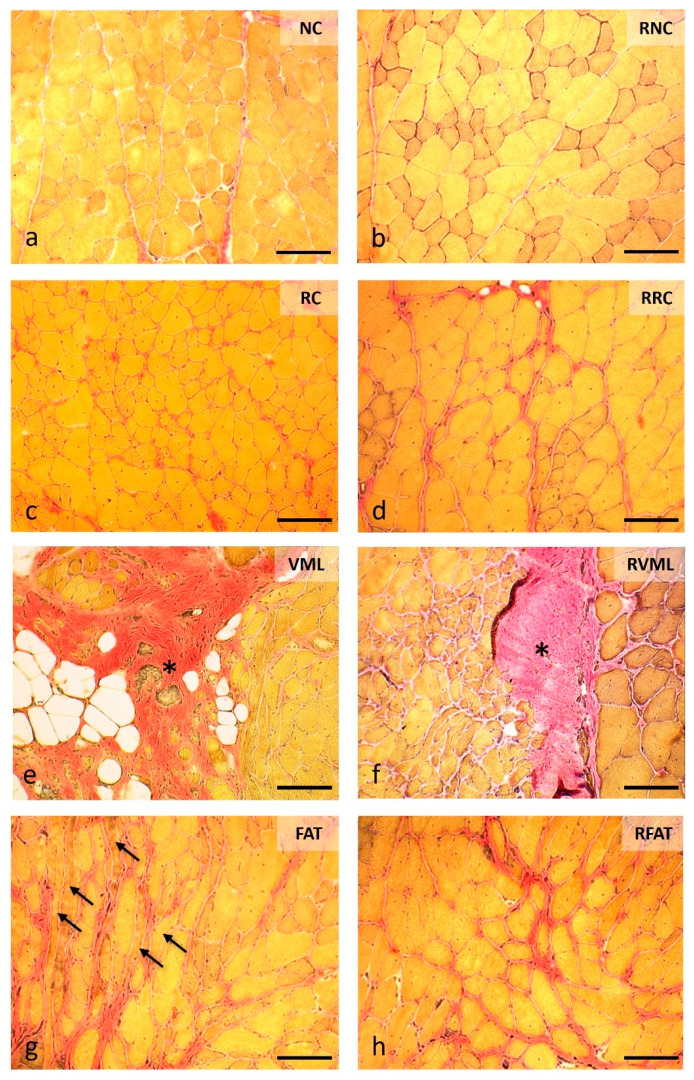
Intramuscular connective tissue content at 60 days in cross-sections stained with Van Gieson’s Trichrome (VGT) in the different experimental groups. (**a**) Endomysium connective tissue is very inconspicuous, only the perimysial connective tissue is partially shown stained in red. (**b**) RNC muscle with normal appearance of intramuscular connective tissue. (**c**) Significant increase in endomysial connective tissue surrounding regenerated muscle fibers, characterized by their internal nuclei. (**d**) An increase in connective tissue more evident at perimysium level. (**e**) The area of the defect is occupied by abundant fibroadipose tissue (asterisk) while at the edge of the lesion muscle fibers of variable size and central nuclei are observed. (**f**) The fibrotic scar (asterisk) is smaller in size and is surrounded by abundant muscle fibers of very variable size and with internal nuclei. (**g**) Area of fibrosis manifested by thickened red bands at the perimysium level surrounding numerous clearly disoriented muscle fibers (arrows). (**h**) Image like the previous one in a RFAT muscle, showing fibrosis located preferably at the perimysium level. Size scale: 100 µm. See Figure 3’s legend for keys.

Compared with the remaining groups, increased perimysial fibrosis was evident in the VML, RVML, FAT, and RFAT groups (Figure 8).

**Figure 9:**
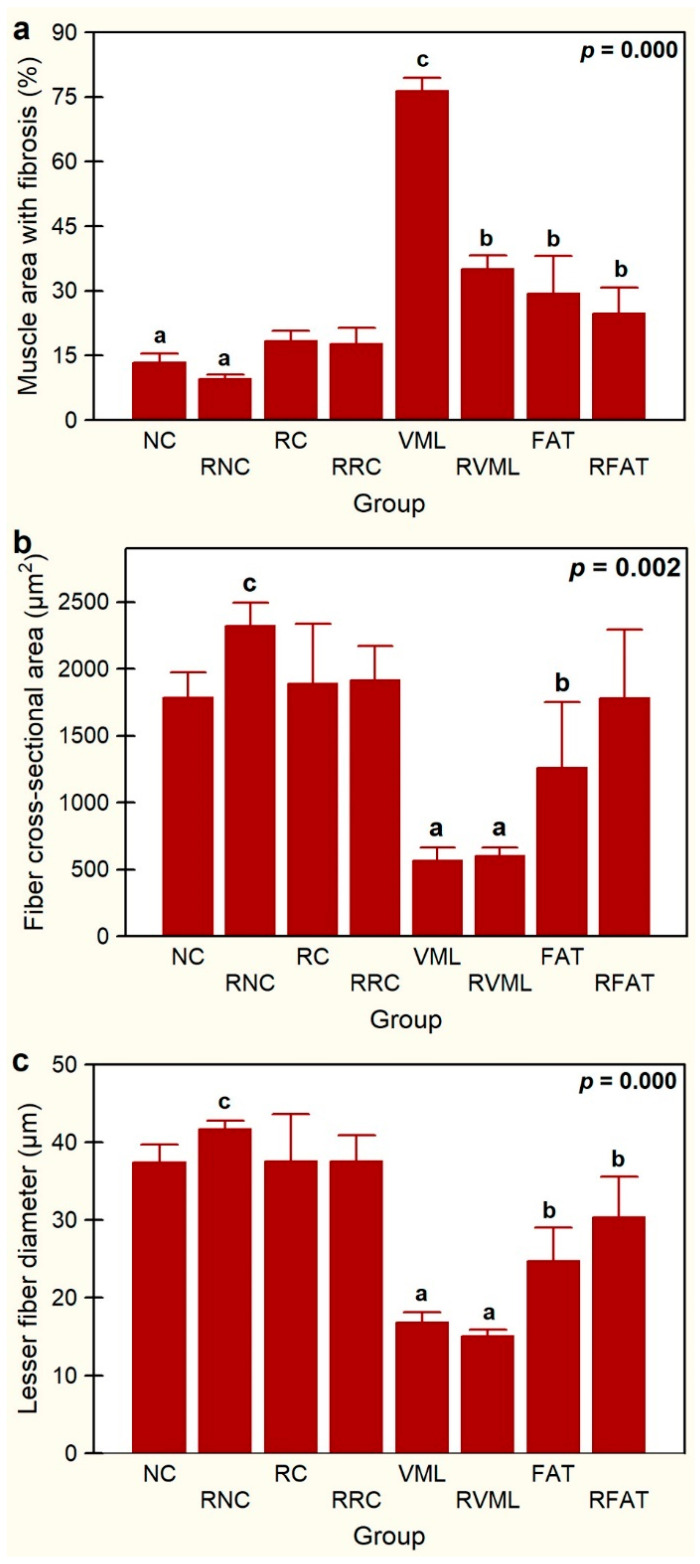
Percentage of muscle area with fibrosis (**a**), fiber cross-sectional area (**b**), and lesser fiber diameter (**c**) in the experimental groups. Data are expressed as mean ± SD of 5 rats per group. *p* values of a nonparametric Kruskal–Wallis h test followed by a Mann–Whitney U test for 2 × 2 comparisons between groups). Means with different lowercase letters are statistically significant (*p* < 0.05 at least). See Figure 3’s legend for keys.

Significant variations in intramuscular fibrosis were also observed among experimental groups, with the VML group showing the largest area of the sample occupied by fibrosis (76.5 ± 3.1%) (Figure 9a). The tested exercise-rehabilitation program did have significant impact on this parameter since the muscle area with fibrosis was reduced by 54% (*p* = 0.000) in the RVML group. The FAT group also showed increased fibrosis, expressed by a greater intramuscular area occupied by connective tissue when compared to both the NC (55%, *p* = 0.03) and RNC (67%, *p* = 0.0001) groups. Similarly, rats in the RFAT group also showed a larger area of fibrosis than RNC rats (61%, *p* = 0.005). As expected, the muscle area with fibrosis was 61% lower in FAT rats than in control VML rats (*p* = 0.000), but the rehabilitated-exercise program did not significantly affect this reduction. Thus, although the area of fibrosis was 16% smaller in the RFAT group versus the FAT group, this difference did not reach the pre-established level of statistical significance (*p* = 0.089).

Globally, significant differences in mean fiber size (CSA, *p* = 0.002 and LFD, *p* = 0.000) were observed between rehabilitated vs non-rehabilitated groups, but individually, there were no significant differences between rehabilitated animals and their non-rehabilitated controls (Figure 9b,c). The animals that suffered muscle injury with volumetric loss and were transplanted with autologous adipose tissue (FAT group) showed muscle atrophy with respect to the RNC group (CSA, −46%, *p* = 0.012; LFD, −41%, *p* = 0.002) (Figure 9b,c). The RFAT group also showed muscle fiber atrophy compared to RNC rats (LFD, −27%, *p* = 0.030), and although the mean fiber size of the RFAT group tended to be higher than in the FAT group, these differences were not statistically significant (*p* > 0.05). Importantly, animals that suffered a muscle injury with volumetric loss and were not transplanted with autologous adipose tissue (VML group), showed a more intense muscle atrophy with respect to the RNC group (CSA, 70%; LFD, 60%; *p* = 0.000) than rats with the same injury transplanted with autologous adipose tissue (FAT group) as expressed above.

**Figure 10:**
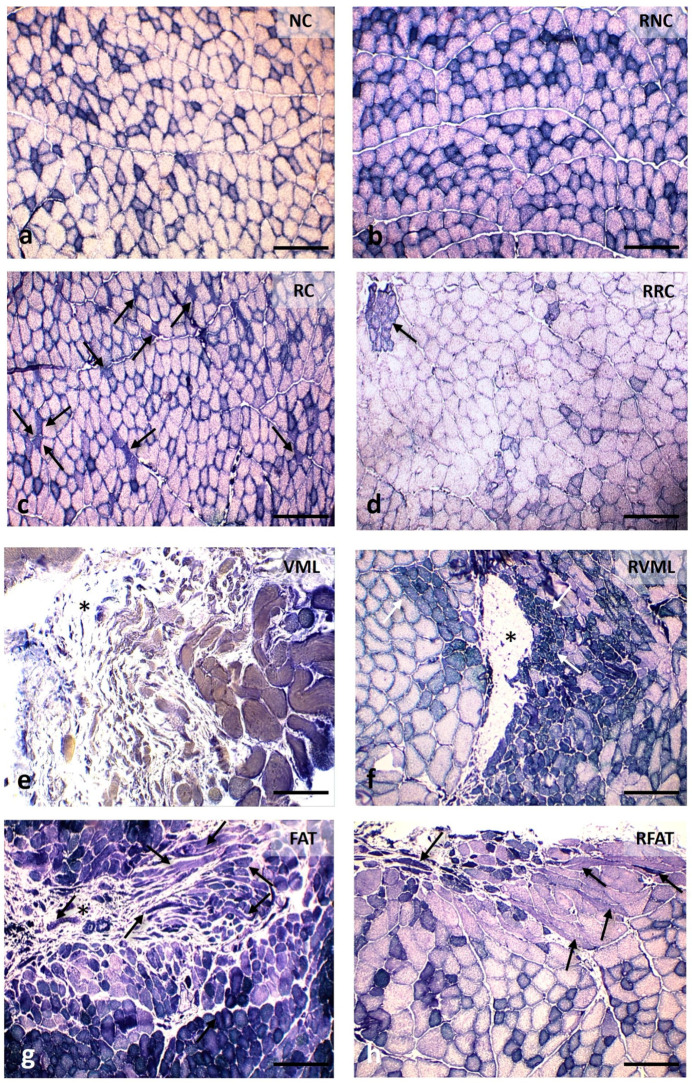
Histochemical changes observed at 60 days in cross-sections stained with the nicotinamide adenine dinucleotide tetrazolium reductase (NADH-tr) reaction in the different experimental groups. (**a**) Normal skeletal muscle showing the characteristic mosaic pattern distribution of muscle fiber types. (**b**) The distinction between muscle fiber types is maintained. (**c**) Several atrophic muscle fibers with an angular profile (arrows). (**d**) Loss of the mosaic pattern with fiber type grouping. While most of the image is practically occupied by large glycolytic fibers, on the left there is a small cluster of oxidative fibers or type I, interpreted as a clear sign of reinnervation. (**e**) Muscle fibers at the edge of fibrosis (asterisk) are highly atrophic, disoriented, and undifferentiated into histochemical types. (**f**) The fibrotic scar (asterisk) is surrounded by muscle fibers of variable size with a clear differentiation of histochemical types, although groupings of the same type are evident (arrows). (**g**) FAT muscle in which, together with a small fibrotic scar (asterisk), a large area of newly formed muscle fibers of variable size and with differentiation of histochemical types of fibers are observed, even in those that are disoriented (arrows). (**h**) The distinction between fiber types is somewhat more difficult than in the previous FAT image, but a normal oxidative profile is recovered even in the disoriented muscle fibers (arrows). Size scale: 200 µm. See Figure 3’s legend for keys.

Histochemical analysis with NADH-tr staining revealed a recovery of the normal oxidative staining pattern of myofibers in the RFAT group, as well as signs of reinnervation in the RRC group (Figure 10).

## 4. Discussion

This study examined the effect of treadmill exercise on the rehabilitation of rat skeletal muscles with a VML lesion reconstructed with autologous adipose tissue. The primary results obtained show evidence that physical exercise has significant effects on certain histoarchitectural parameters. Compared with its matched control group, the scheduled exercise rehabilitation program tested in this study improved both (i) the spatial organization and (ii) the cross-sectional shape of muscle fibers in the RFAT group; it also showed a clear trend to favor the migration of myonuclei towards the cell periphery, the hypertrophy of muscle fibers, and the recovery of the normal oxidative staining pattern. However, it did not ameliorate the intense endo- and perimisial fibrosis observed in muscles with VML injury (e.g., no significant differences in the percentage of muscular fibrosis between FAT and RFAT muscles were observed; see Figure 9). Despite not being the main focus, the results also indicate that the rehabilitation exercise program tested in the present study improves muscle regenerative capacity, favoring the migration of internal nuclei towards the myofiber periphery and promoting signs of reinnervation in the RRC group compared to its matched control group (i.e., RC).

The most notable findings of the present study were the significant reduction in the number of misoriented fibers and the significant normalization of exercise-induced cellular circularity in muscles from VML rats transplanted with autologous adipose tissue. As the experimental design included a control group of rats with VML without autologous adipose tissue transplant subjected to exercise, it can be interpreted that these effects are not attributed to the influence of exercise per se but, alternatively, to the additive interaction between exercise and autologous adipose tissue transplantation.

The benefits of exercise on the ECM during muscle regenerative processes, favoring the correct spatial organization of myofibers [20,21] and reducing fibrosis [22,23,24], are also well known. These effects were partially confirmed in the present study. Our results showed greater fibrosis in VML lesions reconstructed with adipose tissue than in normal and regenerative controls, regardless of whether they had been, or had not been, exercised. The excess of connective tissue observed in these lesions is linked to the intense and chronic inflammatory response that occurs simultaneously [25,26,27], causing a cellular and molecular imbalance that promotes fibrosis, hindering muscle regeneration [28,29,30], and reinnervation of regenerated muscle fibers [31]. Some antifibrotic measures (inhibitors of profibrotic cytokines and growth factors) have had paradoxical effects such as increased inflammation or inhibition of regeneration [5,30]. Therefore, instead of antifibrosis interventions, measures aimed at modulating the extensive inflammatory response present in VML lesions seem more indicated. Lack of physiologic responses to exercise and rehabilitation in muscle VML injuries is clear clinically, but the mechanisms limiting these responses are unclear. The muscle-specific and whole-body metabolic consequences and the chronic loss of function following VML injury may explain the limited regenerative response to rehabilitation in the remaining muscle after VML [32].

Depending on its characteristics (nature and intensity) and the degree of existing inflammation, chronic exercise counteracts the imbalance caused by chronic inflammation [33]. However, compared to sedentary controls, it also promotes collagen synthesis under certain circumstances, such as the presence of exercise-induced microtrauma [34] as well as in VML injury’s scars [8]. This effect leads to a state of “functional fibrosis” that favors the transmission of passive or elastic force, without an intrinsic increase in active or contractile force [26,35].

The specialized literature considers physical exercise as a beneficial therapy for the functional recovery of skeletal muscles with VML injuries [8,23,26,36]. The use of combined regenerative–rehabilitative therapies, such as those tested in this study, improve muscle strength and recovery [13,22] only when there is significant muscle regeneration [26,37]. A trend for this improvement was also observed in the rehabilitated groups of the present study; nevertheless, our results showed that, despite exercise, muscles with VML lesions and transplanted with autologous adipose tissue did not recover the baseline strength of normal muscles, in agreement with previous studies [37,38]. However, the methodology used in the present study may be limited for evaluating functional recovery of VML muscle with autologous adipose tissue transplantation. Thus, the functional test used to assess muscle grip strength appears to primarily involve the plantar flexor muscles, whereas the injured muscle acts synergistically with the dorsiflexor muscles. This apparent incongruity should be resolved in future studies by specifically examining the functionality of the injured muscle, for example via electromyography. Furthermore, due to these methodological limitations, the absence of significant variations in muscle function between the three injury models used in the present study (Figure 5a) does not mean that the VML injury model cannot induce a permanent loss of function in the affected muscle.

It is well known that the structure and function of skeletal muscle depend on both myogenic and neuronal factors [39]. Thus, scheduled physical exercise promotes normal skeletal muscle hypertrophy [26], an effect that has been globally observed in the four groups of rehabilitated animals tested in the present study. The present study has confirmed that the implantation of autologous adipose tissue in VML injuries produces a significant muscle fiber hypertrophy [12]. An exercise-associated increase in myofiber has also been reported in regenerated muscle [23,40], including those with VML lesions treated with combined regenerative–rehabilitative methods [23,37]. The influence of exercise on the size of muscle fibers is controversial, since the results of some previous studies suggest that exercise could favor the proliferation and activation of satellite cells rather than the hypertrophy of mature muscle fibers [34,36].

Previous studies have shown clear benefits of exercise on the reinnervation of denervated muscles [24,41,42]. Although only qualitatively, histochemical observations from the present study also showed evidence that exercise promotes signs of reinnervation in both rehabilitated regenerative controls (reduction in angulated atrophic fibers and grouping of fiber types) and rehabilitated adipose tissue transplant models (recovery of the characteristic mosaic spatial distribution pattern). Likewise, we observed how the loss of oxidative staining associated with VML lesions with adipose tissue transplantation evolved towards normalization of the oxidative staining pattern of the muscles in the exercised groups, probably mediated by mitochondrial biogenesis in response to the in-creased exercise-induced contractile activity [43]. It is also known that mechanical stimulation promotes angiogenesis [22,40], accelerating muscle regeneration and functional recovery [13,34]. In agreement with previous studies [13,22,41], qualitative observations from the present study also indicated an apparent increase in vascularity in exercised damaged muscles. However, despite the preliminary qualitative observations of the present study, new, more specific quantitative studies are needed to confirm the beneficial effect of exercise on the reinnervation pattern, oxidative histochemical pattern, and capillarization of VML muscles.

The literature consulted does not clearly define the optimal time to start the rehabilitation of VML injuries. In VML injuries treated with regenerative therapies, the maximum benefit of exercise occurs in the first 2 weeks [36]. It seems evident that the precocity of physical rehabilitation [35,41], by progressively increasing activity within limits tolerated by pain [44], allows for the obtainment of the most optimal results. In the present study, due to the great extent of the induced injury and the inflammation caused, physical rehabilitation was not started until day 14, and this long period would explain, at least partially, the lack of a more complete exercise-stimulated muscle recovery. For animal welfare reasons, given the severity of the experimentally induced injuries produced, it was decided to wait 2 weeks to start the exercise program [45]. But immobilization was not absolute during this period, as rats had reduced mobility within their cages. It is important to consider that while functional analyses reflect the strength of the whole muscle, histomorphometric analyses are restricted to the injured area, expecting heterogenous results. Therefore, it would be advisable to incorporate analysis of tissue proximal and distal to the defect in future studies as well as the assessment of other more specific markers to investigate in detail the immune response, myosin isoforms, neuromuscular binding, and vascular and metabolic markers of muscle fibers.

## 5. Conclusions

In conclusion, the results of the present study show that physical exercise on a treadmill significantly improved certain histoarchitectural parameters in a VML lesion model reconstructed with autologous adipose tissue. Specifically, it favors the spatial reorganization and cross-sectional shape of the newly formed muscle fibers and promotes the normal migration of their myonuclei towards the periphery, showing a more efficient and complete muscle regeneration process. Qualitative results also showed, although indirectly, evidence that this intervention also promotes reinnervation and normalization of the oxidative intermyofibrillar and mitochondrial profile of the newly formed fibers during this process. However, the physical rehabilitation plan tested did not induce benefits at the extracellular level, as it did not significantly counteract the intense fibrosis associated with these injuries.

These findings, together with those in the specialized literature, indicate that the starting time, type, intensity, and duration of the physical exercise plan must be readjusted to obtain optimal therapeutic results, as well as the development of new non-traditional rehabilitation models that are adaptable to severely injured muscles such as those with VML injury [26,46]. Therefore, new, more extensive studies are needed, with a larger sample size and different rehabilitation protocols, to clarify the additive influence of exercise, combined with regenerative therapies, on the regeneration of severe muscle injuries.

## Data Availability

The datasets used and/or analyzed during the current study are available from the corresponding author on reasonable request.

## Supplementary Materials

The following supporting information can be downloaded at: https://www.mdpi.com/article/10.3390/jfmk9040188/s1, Figure S1: Schematic diagram of experimental design; Figure S2: Histology of control groups; Figure S3: Histology of exercise-rehabilitated groups.

## Abbreviations

CSA: Cross-sectional area; DisMF: Disoriented muscle fibers; ECM: Extracellular matrix; FAT: Fresh adipose tissue; FF: Form factor; FINuc: Fibers with internal nuclei; HE: Hematoxylin and eosin stain; LFD: Lesser fiber diameter; NADH-tr: Nicotinamide adenine dinucleotide dehydrogenase-tetrazolium reductase; NC: Normal control; RC: Regenerative control; RFAT: Rehabilitated fresh adipose tissue; RNC: Rehabilitated normal control; RRC: Rehabilitated regenerative control; TA: Tibialis anterior muscle; VGT: Van Gieson’s trichrome stain; VML: Volumetric muscle loss.
