# Supplementary material for: Influence of Physical Exercise on the Rehabilitation of Volumetric Muscle Loss Injury Reconstructed with Autologous Adipose Tissue
Source: J Funct Morphol Kinesiol. 2024 Oct 8;9(4):188. doi: 10.3390/jfmk9040188 (PMC11503405; doi:10.3390/jfmk9040188)
Supplement: Supplementary file 1 [file jfmk-09-00188-s001.zip › jfmk-3177926-supplementary.pdf]

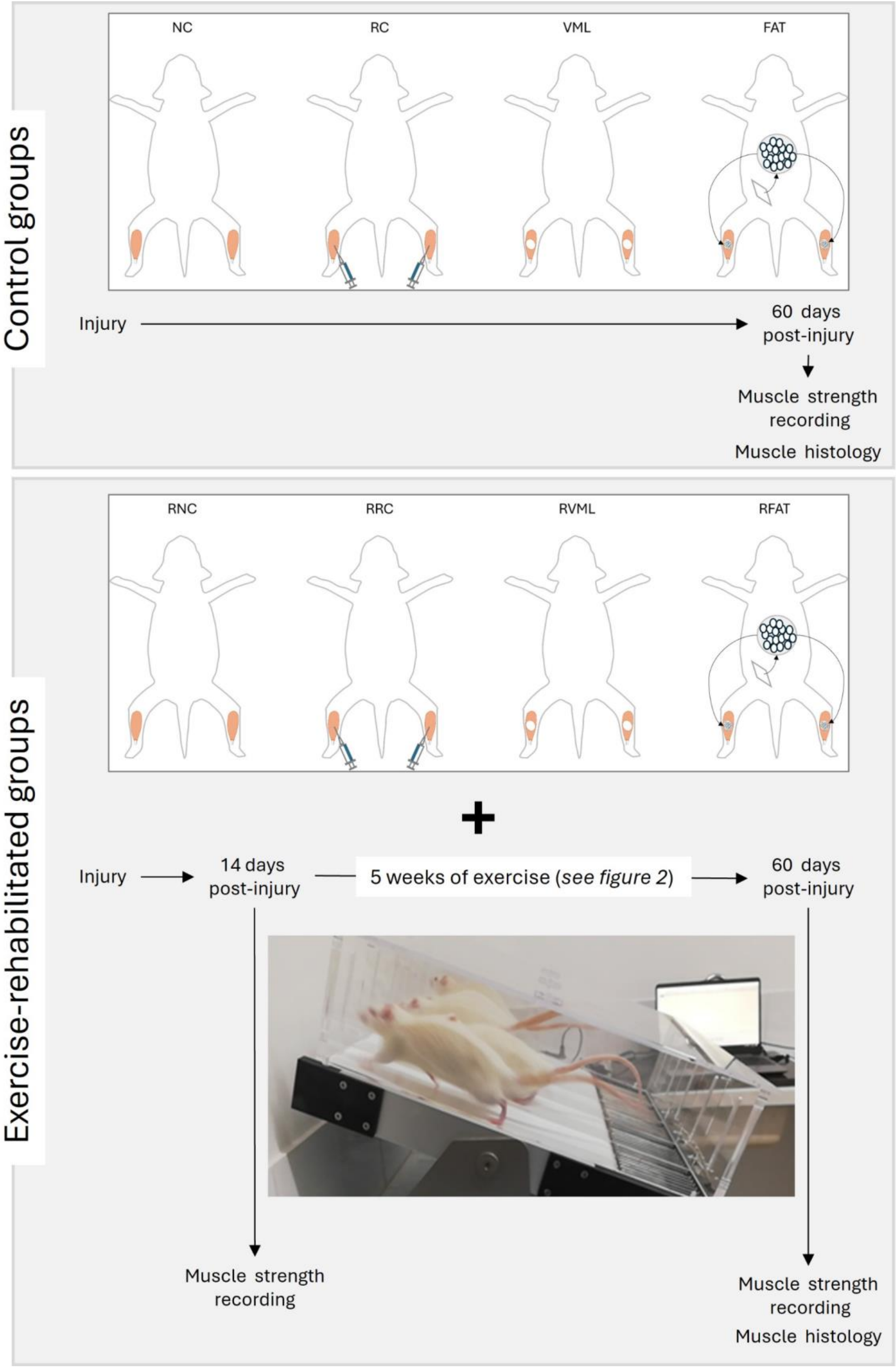

Supplementary figure S1. Schematic diagram of experimental design.

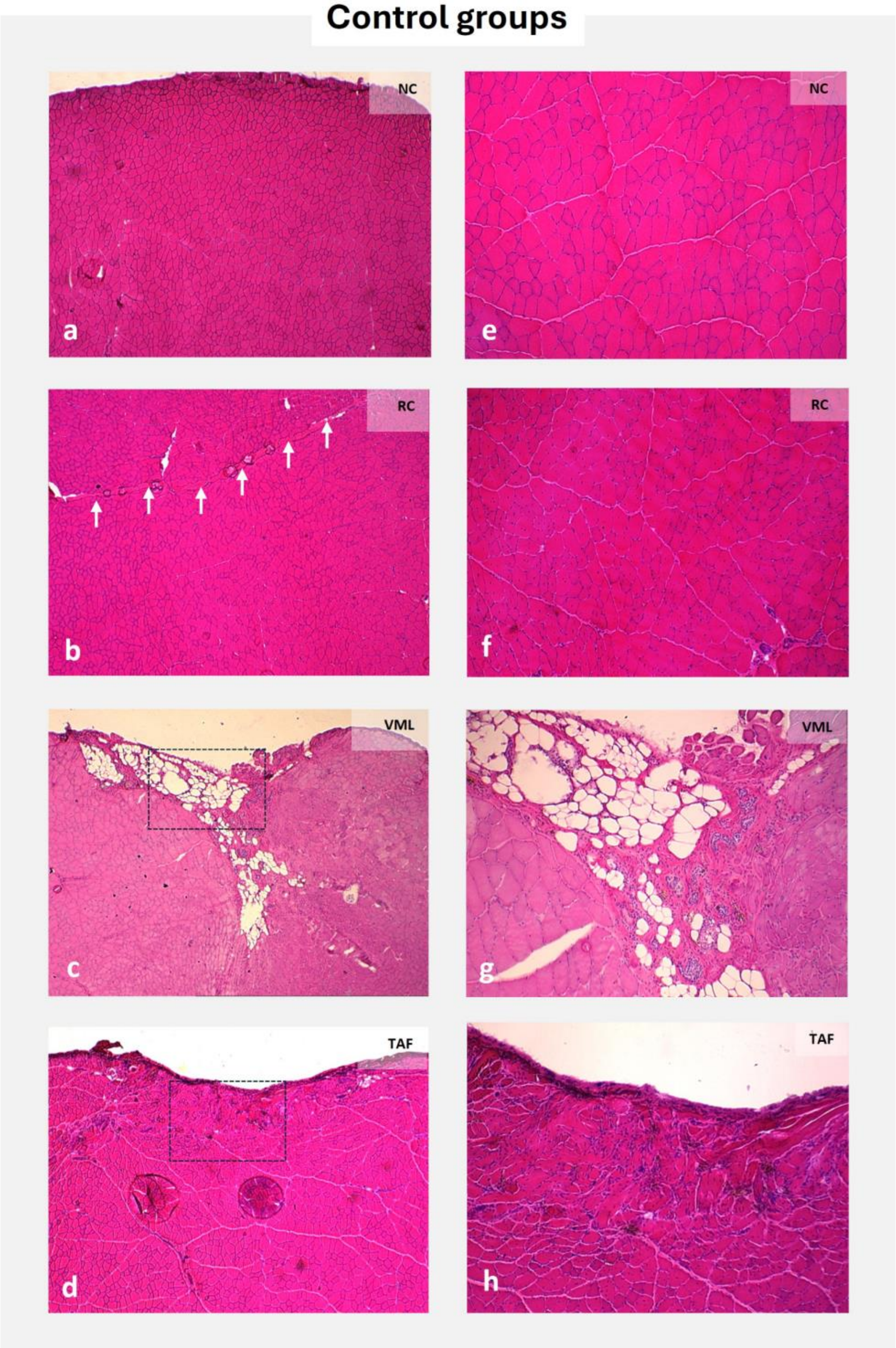

Supplementary figure S2. (a, e) Normal muscle structure. (b, f) The arrows mark a fibrotic band resulting from the intramuscular injection. The organization in muscle fascicles is preserved and the muscle fibers show a normal morphology although most have central or internalized nuclei. (c, g) The lesion area is occupied by abundant fibro-adipose tissue. (d, h) On the surface of the lesion area there is a slight depression, below which we find abundant muscle fibers, although fascicles are not visible; in the enlargement (h) a significant disorientation of the muscle fibers is observed, in addition to an aberrant morphology. Hematoxylin-eosin. a-d, 4x; e-h, 10x.

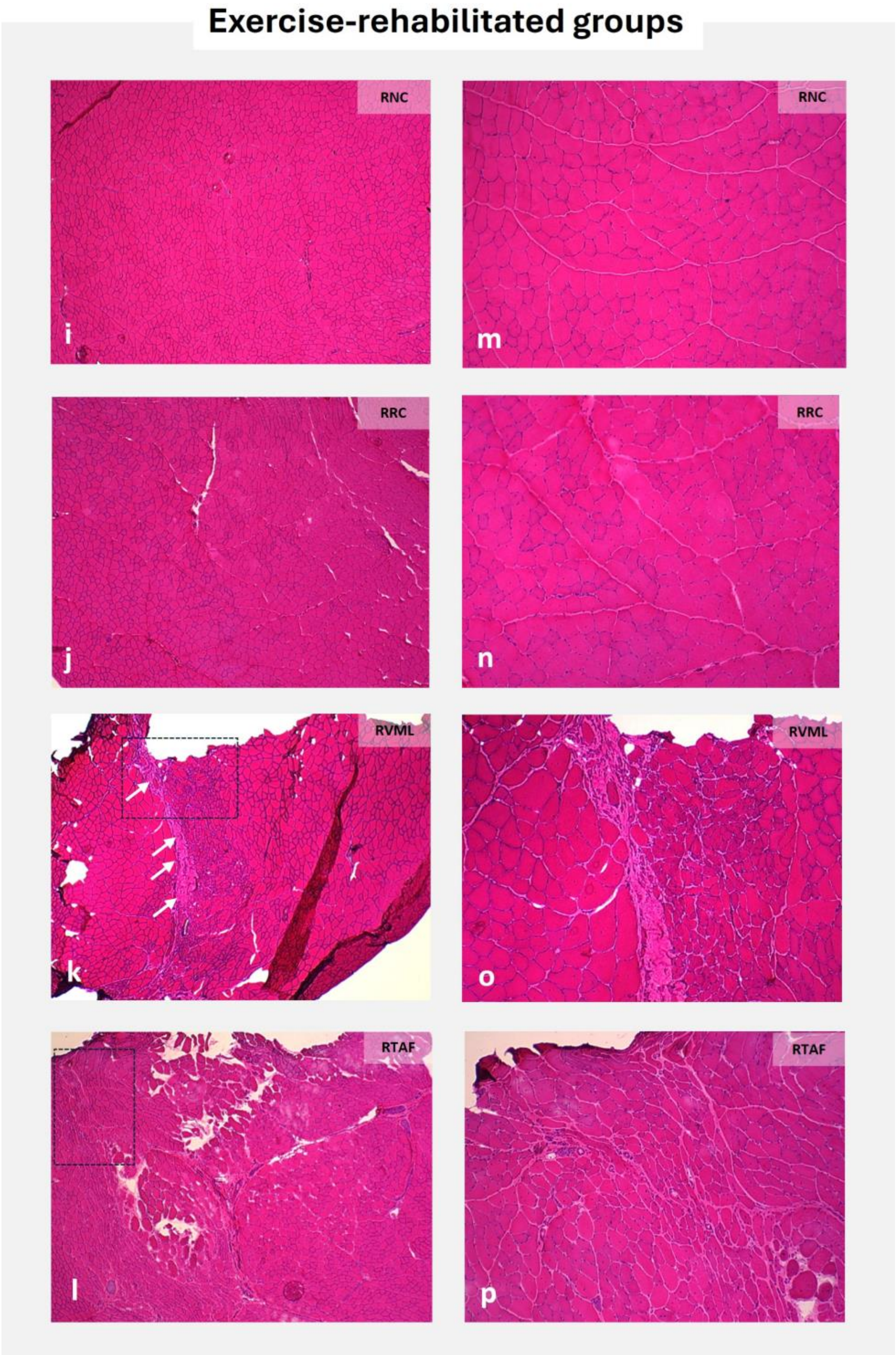

Supplementary figure S3. (i, m) Muscle structure without apparent morphological changes. (j-n) Muscle fibers are grouped in fascicles and show normal morphology and staining; unlike what is seen in images b and f (fig. S2), many muscle fibers do not have central or internalized nuclei. (k, o) The lesion area is seen occupied by a fibrotic band (arrows), next to which regenerated muscle fibers of variable size with internalized nuclei are arranged. (l, p) The lesion area is occupied by skeletal muscle tissue with a certain organization in fascicles between which intramuscular nerves and blood vessels are clearly visible. Muscle fibers contain internal nuclei, are of variable size and, mostly, are well oriented. Hematoxylin-eosin. i-l, 4x; m-p, 10x.
